# App-Based Analysis of Fluoroscopic Images According to Bernard-Hertel Method for the Determination of Femoral Tunnel Positioning in Anterior Cruciate Ligament Reconstruction

**DOI:** 10.1016/j.eats.2023.10.006

**Published:** 2024-01-01

**Authors:** Juergen Hoeher, Oliver Tenfelde, Ben Wagener, Markus Fink, Alejandro Mauri-Moeller, Maurice Balke

**Affiliations:** SPORTSCLINIC COLOGNE, Cologne, Germany; University of Witten-Herdecke, Witten, Germany; Cologne Merheim Medical Center, Cologne, Germany

## Abstract

The accurate positioning of the femoral tunnel is crucial for the success of anterior cruciate ligament reconstruction. Malpositioning of the tunnel is believed to be one of the most important reasons for graft failure. While use of anatomic landmarks and industry-supplied aiming devices aid the surgeon in placing the drill pin in the correct position, fluoroscopic imaging is an additional tool used intraoperatively to verify pin placement. While interpretation of fluoroscopic imaging is frequently based on eyeball measurement, a more accurate analysis of a lateral image uses the quadrant method by Bernard-Hertel. This method has been primarily used for scientific research due to its complexity and has not been integrated into clinical routine yet. We present a digital app-based approach to easily quantify the femoral pin position based on the quadrant method. This approach is mobile and easy to use. Quantification of pin position of femoral bone tunnel on a lateral fluoroscopic image may be used for quality control and teaching purposes or may provide the surgeon with additional information during ACL reconstruction.

## Clinical Background

Accuracy of tunnel placement is crucial for the success of anterior cruciate ligament (ACL) reconstruction. Malpositioning of bone tunnels is considered an important factor for graft failure.^1^, ^2^, ^3^, ^4^ The surgeon performing ACL reconstruction may use anatomic landmarks for orientation during surgery such as the lateral border of the posterior cruciate ligament, the posterior edge of the lateral femoral condyle (over the top), the posterior horn of the lateral meniscus,^5^ or remnants of a ruptured ligament at the femoral insertion site. However, these landmarks are sometimes difficult to define.^6^, ^7^, ^8^ In addition, the arthroscope only provides a 2-dimensional image; therefore, depth relations may be misleading. Usually, industry-supplied aiming devices (so-called offset devices) are used to place a 2.4-mm central pin into the center of the desired location during surgery. Further, portal placement and the amount of knee flexion will have an important impact on the direction of the femoral tunnel positioning.^9^^,^^10^

Besides anatomic landmarks, radiographic analysis was shown to be helpful to define the insertion area of the ACL at the femur.^11^^,^^12^ The quadrant method by Bernard and Hertel^13^ described that the femoral footprint can accurately be defined on a true lateral radiograph. Since the first publication, the quadrant method has been used by many authors mostly for scientific purposes to quantify tunnel positioning in cadaveric studies or after surgery.^14^, ^15^, ^16^, ^17^, ^18^ To quantify a correct pin position according to Bernard and Hertel, additional lines must be drawn on an image to make calculations, so that the relative position of the bone tunnel on the lateral femur can be quantified. These measurements can be made by sheet and pencil or with the aid of various software programs (i.e., canvas). For intraoperative analysis during surgery, Kumar et al.^19^ have suggested to use an indigenously made grid on a transparency sheet to apply a quadrant analysis after fluoroscopic imaging during surgery. All of these methods provide an exact evaluation of the fluoroscopic image; however, they are time-consuming and lack ease of use.

We present a technique in which a lateral radiograph can be digitally analyzed according to the quadrant method by use of a smartphone app.

## Surgical Technique

If fluoroscopic imaging is used during ACL surgery and the image is aimed to be analyzed by the quadrant method, a true lateral radiograph of the knee is required. We have found that it is difficult to take such a radiograph during arthroscopy with the knee in high flexion.^9^ We therefore prefer another approach.

**Fig 1:**
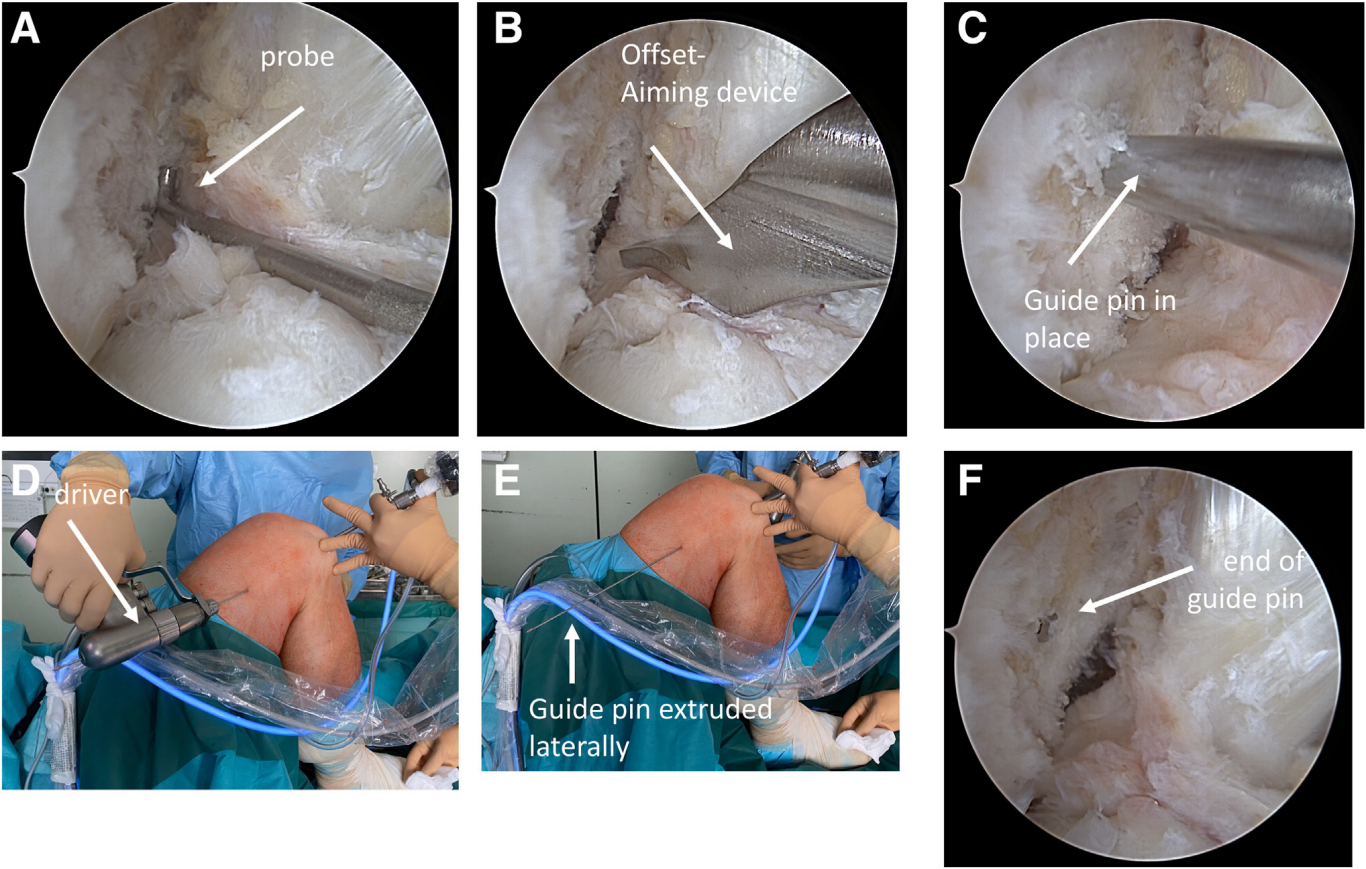
Technique of placing a guide pin into the lateral femoral condyle during anterior cruciate ligament (ACL) surgery and forwarding the pin so that a radiograph can be taken for analysis of its position. (A) Intercondylar view via anterolateral portal of a right knee after preparation of the lateral footprint with the probe at the dorsolateral aspect of the lateral femoral condyle. (B) Identical view with a 7-mm offset aiming device introduced through a low anteromedial portal in 125° of knee flexion. (C) Identical view with guide pin in place after removal of aiming device. (D) The guide pin is driven forward with the driver from the lateral aspect of the knee. (E) The tip of the guide pin can be seen at the lateral aspect of the knee. (F) The back end of the guide pin (red arrow) is flush with the cortex of the femoral condyle within the ACL footprint.

The surgeon should place a guide pin for the femoral tunnel under arthroscopic control in deep knee flexion in the desired way. The guide pin is driven further so that it extrudes the skin on the lateral aspect of the femur. The pin is then driven further after placing the driver on the pin extruding from the lateral side so that its back end is flush with the medial cortex of the lateral femoral condyle. This is confirmed both by arthroscopic view and by probing the cortex at the back end of the pin (Fig 1).

**Fig 2:**
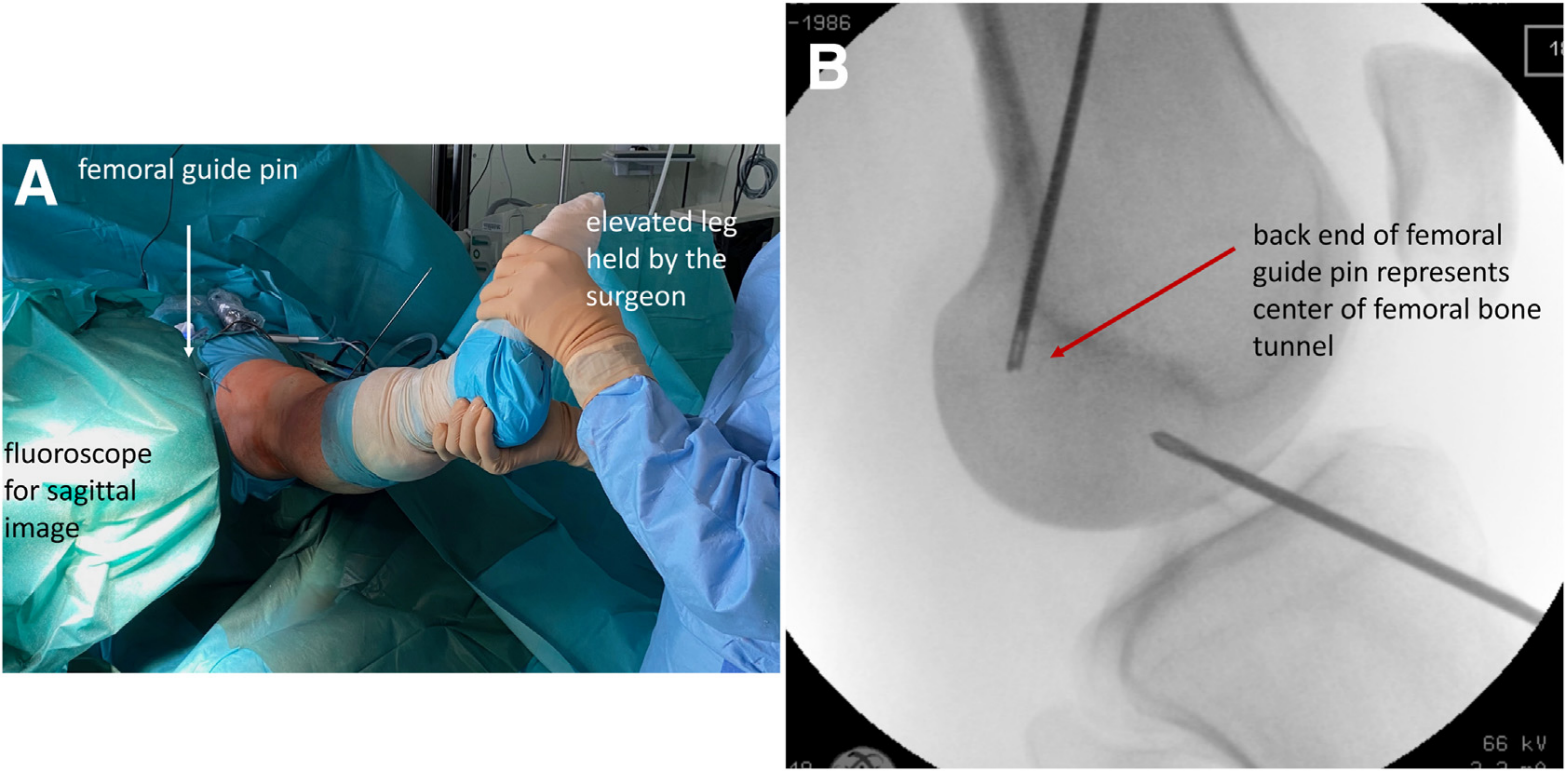
Technique of obtaining a true lateral radiograph with a guide pin in place and positioned so that the end of the pin is flush with the lateral notch cortex. (A) A technique of obtaining a true lateral radiograph in a right knee intraoperatively. The surgeon holds the elevated leg at the foot with the fluoroscope in sagittal orientation By sideway movements and rotation of the leg, a perfect alignment of both condyles can easily be achieved for a true lateral radiograph. (B) Example of a true lateral radiograph with both condyles in perfect alignment. The back end of the femoral guide pin represents the center of the femoral bone tunnel.

Then, all instruments are removed from the joint, and a fluoroscope (C-arm) is moved into the surgical field. The C-arm is brought into a horizontal position. By abducting or adducting the hip joint and also rotating the leg internally or externally, a true lateral image can be obtained and stored on the image intensifier (Fig 2).

After judging the lateral radiograph by eyeball measurement, the surgeon may continue with the ACL surgery. The guide pin is driven backward with the knee in identical flexion as previously. In our hands, a hollow instrument (such as the outer sleeve of an acromionizer/burr) helps to bring the back end of the drill guide back through the anteromedial portal. Then the procedure is continued in a normal fashion by overdrilling the guide pin with a cannulated reamer, and common steps of ACL reconstruction can be continued.

After surgery, the lateral radiograph may be accurately analyzed with use of the smartphone app.

First, the app ACL-X (Linova Corp; available on Apple App Store, https://Apps.apple.com/de/app/acl-x/id1439731734, version 1.0.2 v. 21.05.2020) has to be downloaded (free of charge). The app has to be opened and a security advice has to be confirmed that information from any measurements may not be used for decision-making during the procedure. Then, a picture from a true lateral radiograph is recorded with the digital camera from a printed picture, a computer screen, or a fluoroscopic monitor. As all measurements only display relative distances, no calibration of the picture is necessary.

The picture should be made as such that the outlines of the femoral condyles and Blumensaat’s line are visible at a maximum resolution. After confirming the picture, a rectangular grid can be superimposed on the picture. As a first step, a red line will be established on the screen by touching the smartphone screen with 2 fingers (ideally index and middle finger). This line can be moved and turned in all directions. It should be positioned that it perfectly overlies Blumensaat’s line. By forwarding the app to the next step, the line will be fixed. Now by touching the screen again with 1 finger (ideally just the index finger), a blue line will appear on the screen that is perpendicular to the red line and cannot be turned. It has to be moved so that it is placed at the point where the red line (representing Blumensaat’s line) crosses the posterior cortex of the femur. In the next step, another blue line will appear and is positioned at the red line cutting the anterior cortex of the femur. Next, a yellow line will appear by touching the screen being parallel to the red line. This line has to be placed as a tangent to the most distal part of the femoral condyles. After completing these steps, a rectangular grid has been superimposed to the image. Finally, a bullet eye appears on the screen and has to be moved so that it overlies the tip of the guide pin. By confirming the grid and forwarding the app, 2 numbers are displayed: first, the relative distance of the bull’s-eye position from the posterior condyle line (depth relation in percentages) and, second, the relative distance of the bull’s eye from Blumensaat’s line (height relation in percentages). This image may be uploaded to any destination (e.g., patient chart, surgeon’s folder for teaching or scientific purposes). By closing the app, the final image (with the values of the measurement) will be stored. The image is tagged by date, time, and second without any personal data. It can be retrieved from the app any time later.

**Fig 3:**
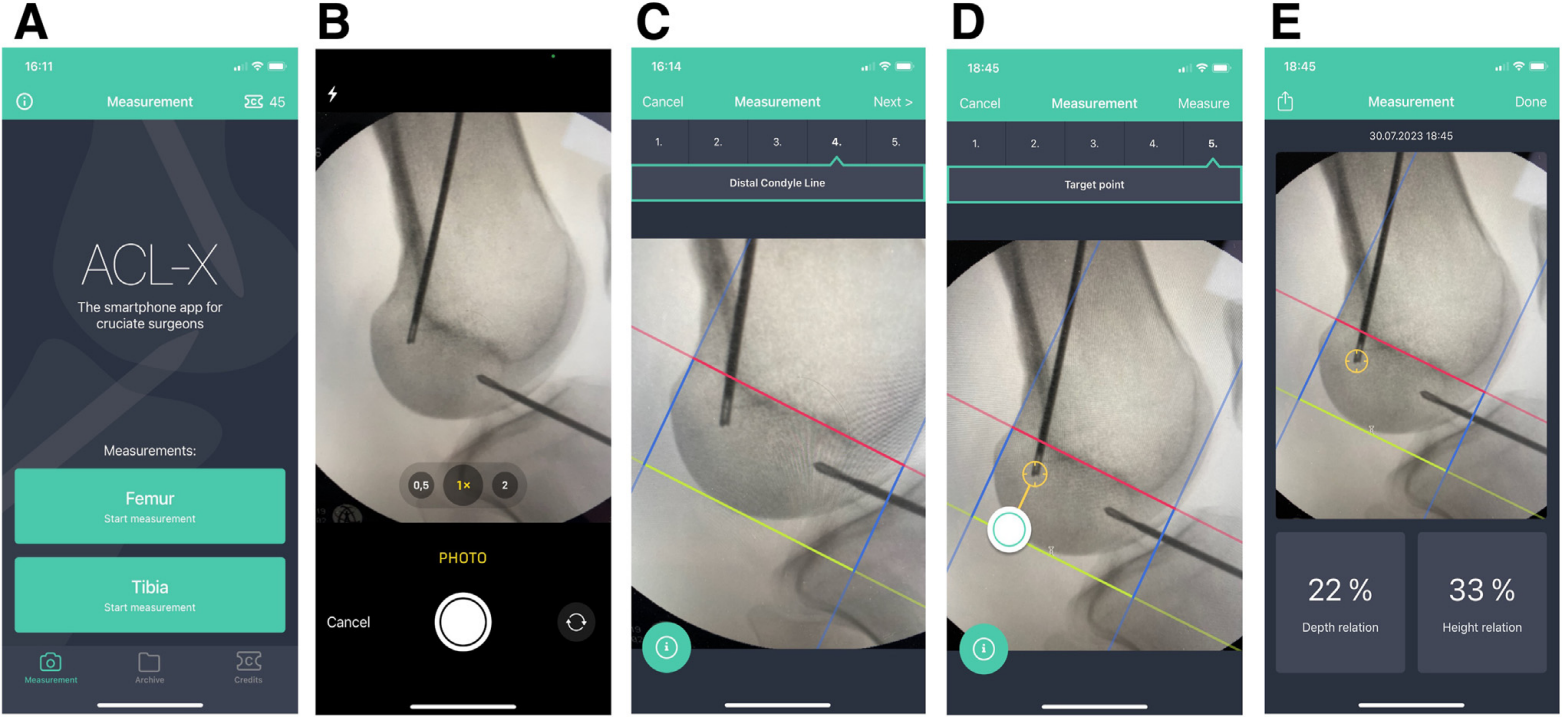
Steps for digital analysis of a true lateral radiograph using the ACL-X app. To perform this analysis, the back end of the guide pin has to be forwarded to the femoral tunnel entrance. (A) Title screen of the ACL-X app (download from Apple App Store: https://Apps.apple.com/de/app/acl-x/id1439731734, version 1.0.2 v. 21.05.2020). (B) Step 1: Open ACL-X app on your iPhone: Take a picture of a true lateral radiograph from the fluoroscopic monitor or from a computer screen and confirm the picture (you can retake the picture if it is not suitable). (C) Steps 2-5: Establish a line on the screen (in red) by touching the smartphone screen with 2 fingers (ideally index and middle finger) and move and turn the line so that it perfectly superimposes Blumensaat’s line. Confirm this position by pushing “next.” By touching the screen with 1 finger (usually index finger), a second line on the screen (in blue) is established. The line will be perpendicular to Blumensaat’s line. Move the line with your finger so that in crosses the endpoint of the red line at the most posterior edge of the femoral condyle. The line is confirmed by pushing “next.” By touching the screen with 1 finger again, another line (in blue) is established on the screen (also perpendicular to the red line). Move the line with your finger so that it crosses the endpoint of the red line at the most anterior edge of the femur. Confirm the line by moving to the next step. By touching the screen with 1 finger, another line (in green) is established on the screen (parallel to the red line). Move the line with your finger so that it runs as a tangent to the most distal part of the femoral condyle. Confirm the position by moving to the next step. (D) Step 6: By touching the screen with 1 finger, a bullet point appears on the screen. Move the target so that its center (bull’s eye) perfectly superimposes the back end of the guide pin on the underlying image. (E) Step 7: The app will automatically calculate the relative distance of the center of the target (bull’s eye) from the posterior condyle line (depth relation) and from the Blumensaat’s line (height relation) in percentages from the total grid’s depth and height. Both numbers are displayed on the screen. This image will be automatically saved in the archive of the app and can be retrieved any time later. In addition, it may be uploaded to another destination (such as patient’s file for documentation or surgeon’s file for personal use).

The use of the app is summarized in Figure 3. Further, in Video 1, the use of the ACL-X app is demonstrated in detail as a tutorial.

## Discussion

Locating the ideal femoral tunnel position is a major challenge for the surgeon during ACL reconstruction. Even though surgical techniques and instruments have advanced in recent years, there are still certain pitfalls depending on technique, portal view, and knee flexion. Tunnel malpositioning might occur especially for low-volume surgeons with less experience.^20^ Fluoroscopic control of the tunnel position has proven to be a helpful tool in confirming correct positioning,^21^ but a user-friendly and time-saving method has been lacking so far.

The use of ACL-X represents an easy way to measure the femoral pin position on a lateral radiograph using a smartphone app. The app leads the user through 6 steps to evaluate femoral pin positioning based on the quadrant method, which still seems to be the gold standard nowadays. It is quick and easy to use, and it can be performed by qualified personal with a short learning curve. Even more, the method creates a digital image file that can be transferred easily to a patient folder or picture archiving system.

Even though the technique offers some advantages, there are also limitations to it (Table 1). To perform the measurement via the app correctly, a true lateral image of the knee is necessary and therefore radiation exposure to the surgical team and the patient is inevitable. The use of the C-arm is time-consuming, but time and the necessary shots to get a true plane picture might go down with experience and procedure routine. The quadrant method has good reliability and low interobserver variability, but the presented variation has not been validated yet. There is concern that measurements might not be reproducible and reliable. Future research should aim to further validate this approach.

**Table 1:** Pearls and Pitfalls of Smartphone App ACL-X

Pearls	Pitfalls
Reproducable quantified measurement of quadrant method quickly and mobile	Requires lateral fluoroscopic image
Short learning curve to qualified personal	Radiation exposure and additional surgery time
Supports the surgeon to find ideal positioning for femoral bone tunnel	Reliability and interobserver variability not known
Helpful for teaching and scientific purposes	Correlation of quadrant method to clinical outcome is not confirmed
Easy way to document correct tunnel position	

## Disclosure

The authors report the following potential conflicts of interest or sources of funding: J.H. is the originator and copyright holder of the ACL-X app, receives financial benefits from user fees for using the ACL-X app in the Apple store, and is a paid consultant for Richard Wolf Corp. Knittlingen, Germany, and OPED Corp., Valley, Germany. All other authors declare that they have no known competing financial interests or personal relationships that could have appeared to influence the work reported in this paper*.* Full ICMJE author disclosure forms are available for this article online, as supplementary material.

## Supplementary Data

ICMJE author disclosure forms

Video 1 In this video, the application of ACL-X (download from Apple App Store: https://Apps.apple.com/de/app/acl-x/id1439731734, version 1.0.2 v. 21.05.2020) is explained step by step on a screen video: a surgeon demonstrates how the smartphone application ACL-X can be used to analyze a true lateral radiograph of the knee with the back end of a guidewire placed into the center of projected femoral bone tunnel (note: in order to use the app, the guidewire has to be forwarded to a position so that the back end of the guidewire is flush with the medial cortex of the lateral femoral condyle).
